# Supplementary figures and images for: Mating strategy predicts gene presence/absence patterns in a genus of simultaneously hermaphroditic flatworms
Source: Evolution. 2022 Oct 31;76(12):3054–66. doi: 10.1111/evo.14635 (PMC10092323; doi:10.1111/evo.14635)

A) Testis region

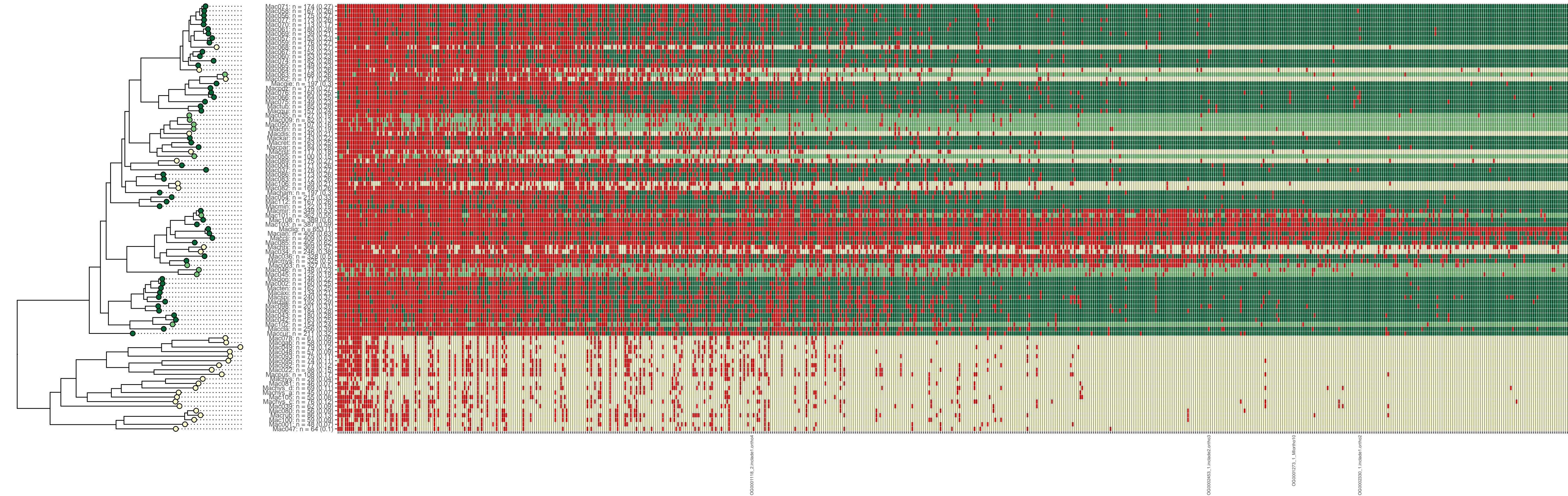

Supplement: Supplementary file 1 — Supplementary figureS1A [file EVO-76-3054-s010.pdf]

B) Ovary region

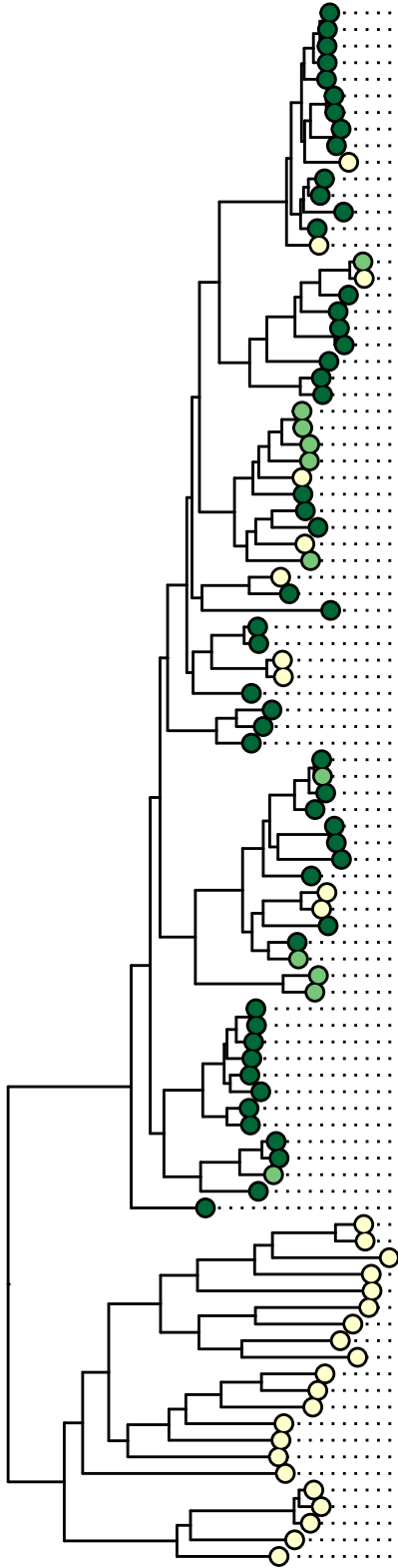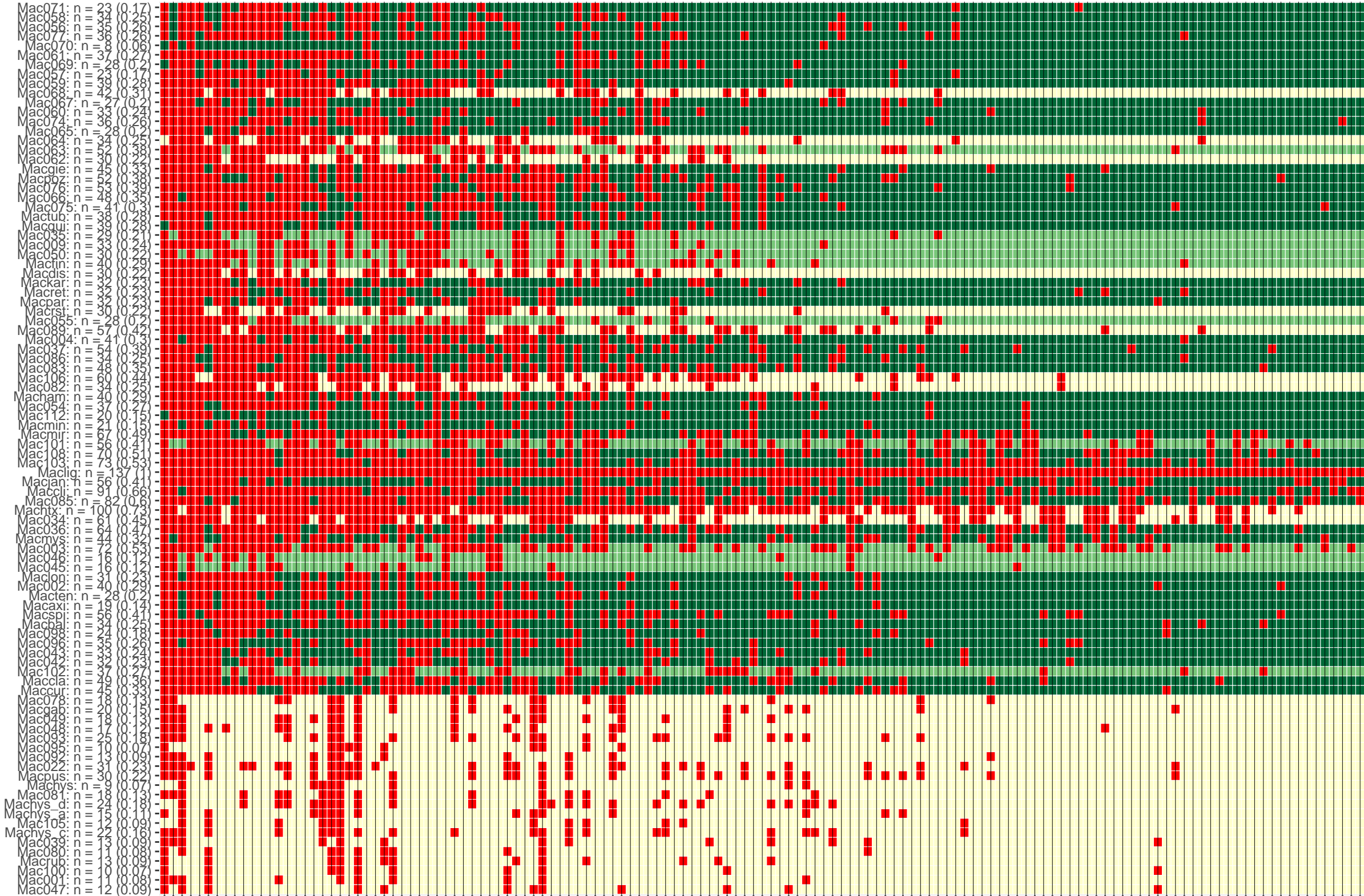

Supplement: Supplementary file 2 — Supplementary figureS1B [file EVO-76-3054-s007.pdf]

### C) Tail region

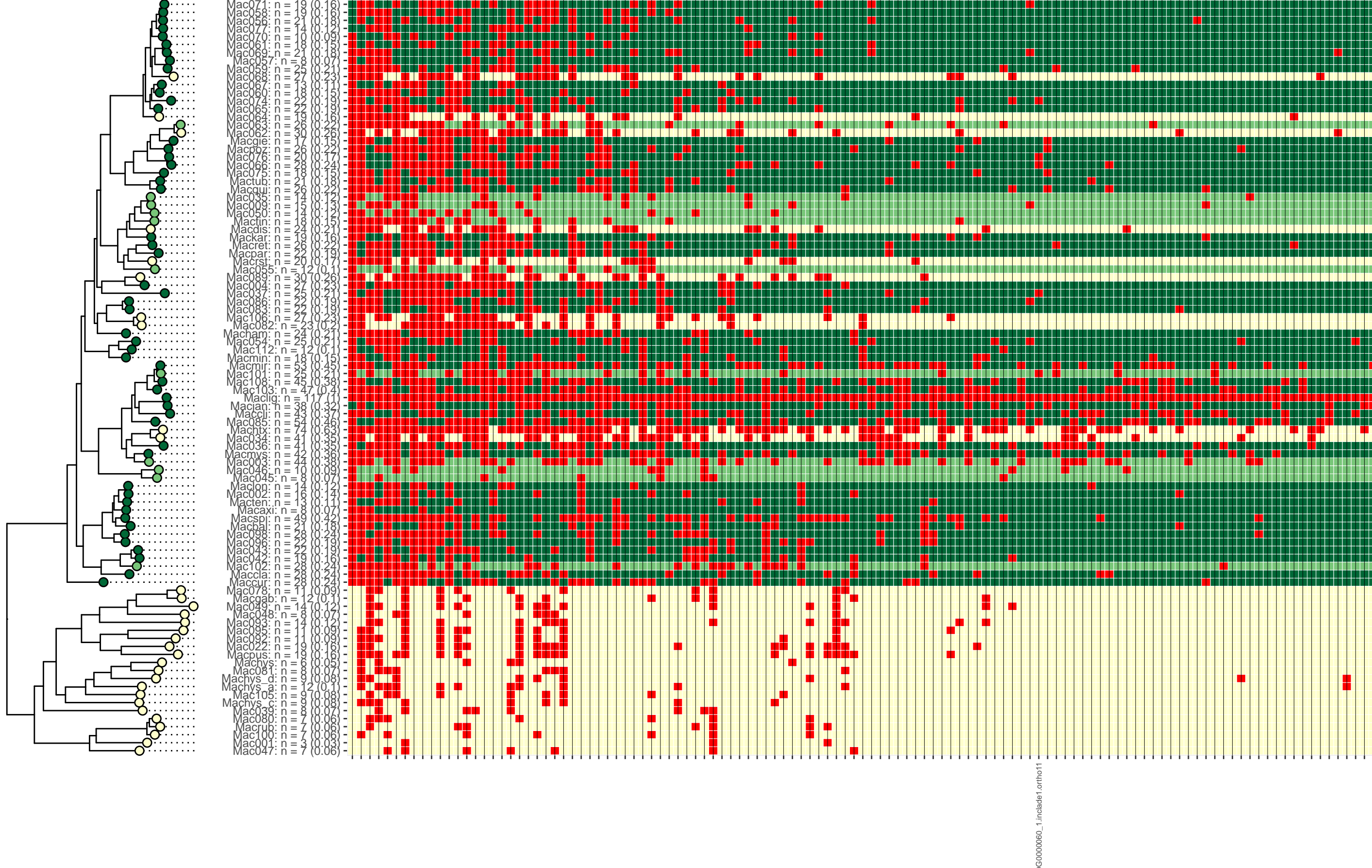

Supplement: Supplementary file 3 — Supplementary figureS1C [file EVO-76-3054-s001.pdf]

D) Ubiquitously expressed

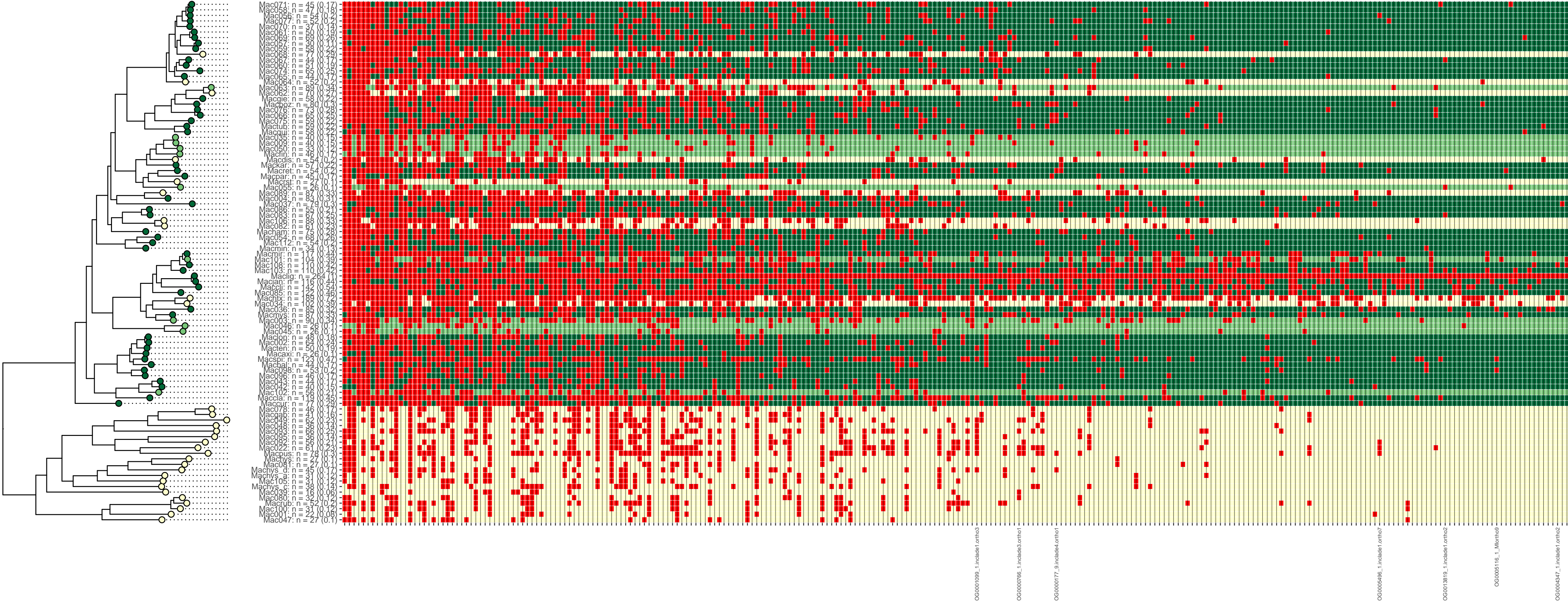

Supplement: Supplementary file 4 — Supplementary figureS1D [file EVO-76-3054-s006.pdf]

OGs with a representative sequence (%)

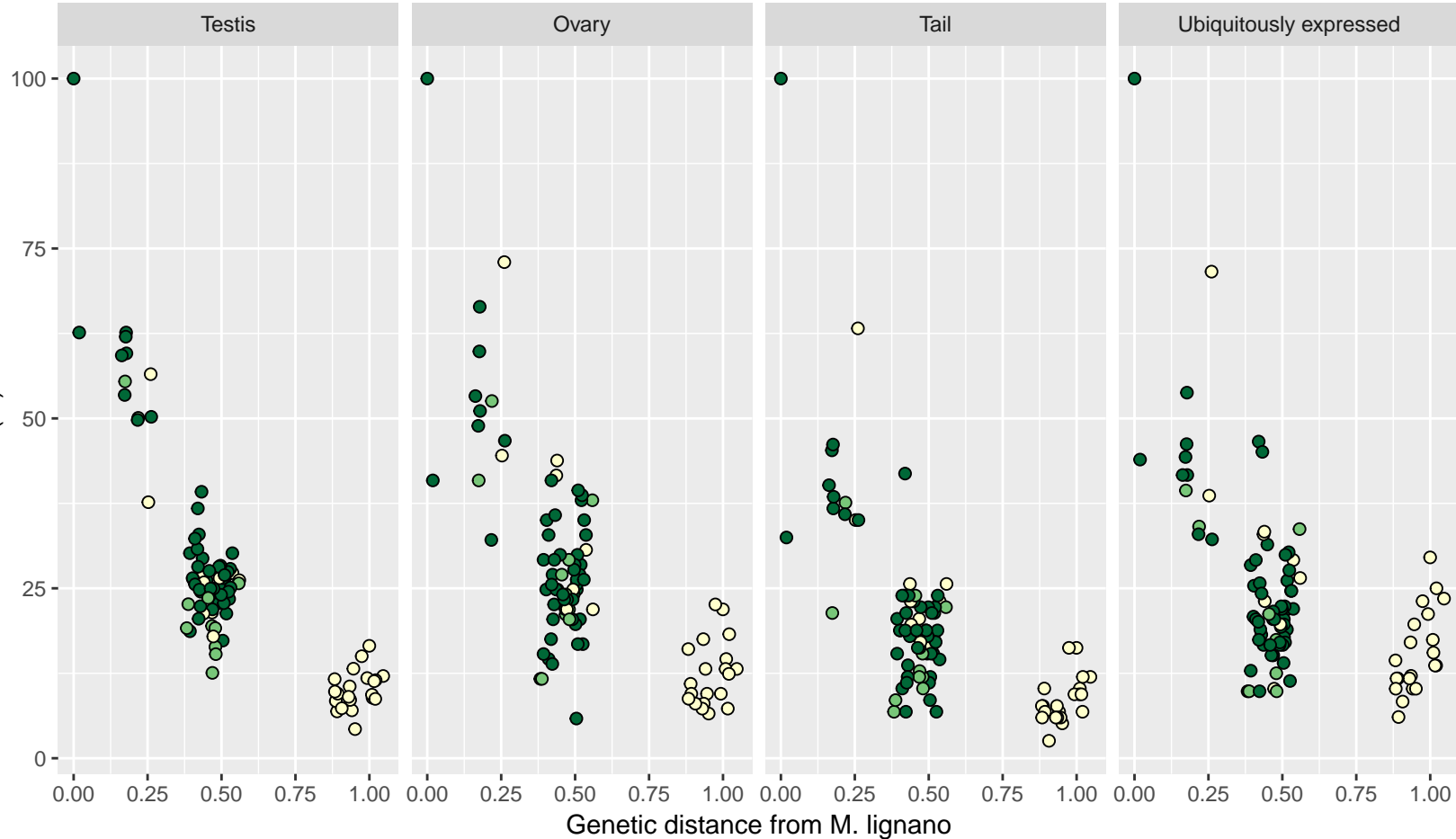

Supplement: Supplementary file 5 — Supplementary figureS1E [file EVO-76-3054-s005.pdf]
